# Evaluation of Possible Effects of a Potassium Channel Modulator on Temporal Processing by Cochlear Implant Listeners

**DOI:** 10.1007/s10162-018-00694-z

**Published:** 2018-09-19

**Authors:** Robert P. Carlyon, John M. Deeks, François Guérit, Wiebke Lamping, Alexander J. Billig, Charles H. Large, Shakeel R. Saeed, Peter Harris

**Affiliations:** 1 Medical Research Council Cognition and Brain Sciences Unit, University of Cambridge, 15 Chaucer Road, Cambridge, CB2 7EF UK; 2 Autifony Therapeutics Limited, Imperial College Incubator, London, UK; 3 Royal National Throat, Nose and Ear Hospital, UCL Ear Institute, 330 Gray’s Inn Road, London, WC1X 8DA UK

**Keywords:** temporal processing, AUT00063, kv3.1, cochlear implant (CI), rate discrimination ratio (RDR), gap detection threshold (GDT), midpoint comparison procedure (MPC)

## Abstract

Temporal processing by cochlear implant listeners is degraded and is affected by auditory deprivation. The fast-acting Kv3.1 potassium channel is important for sustained temporally accurate firing and is also susceptible to deprivation, the effects of which can be partially restored in animals by the molecule AUT00063. We report the results of a randomised placebo-controlled double-blind study on psychophysical tests of the effects of AUT00063 on temporal processing by CI listeners. The study measured the upper limit of temporal pitch, gap detection, and discrimination of low rates (centred on 120 pps) for monopolar pulse trains presented to an apical electrode. The upper limit was measured using the optimally efficient midpoint comparison (MPC) pitch-ranking procedure; thresholds were obtained for the other two measures using an adaptive procedure. Twelve CI users (MedEl and Cochlear) were tested before and after two periods of AUT00063 or placebo in a within-subject crossover study. No significant differences occurred between post-drug and post-placebo conditions. This absence of effect occurred despite high test-retest reliability for all three measures, obtained by comparing performance on the two baseline visits, and despite the demonstrated sensitivity of the measures to modest changes in temporal processing obtained in other studies from our laboratory. Hence, we have no evidence that AUT00063 improves temporal processing for the doses and patient population employed.

## Introduction

Despite the success of cochlear implants (CIs) in restoring hearing to more than half a million people worldwide, auditory perception by CI listeners suffers from fundamental limitations in spatial selectivity and in temporal processing compared to normal hearing (NH). Both of these can be revealed using psychophysical and physiological experiments in which simple stimuli are presented to one or more CI electrodes.

Limitations in spatial selectivity are reflected in the broad spread of neural excitation resulting from stimulation of a single electrode, as measured both using psychophysical techniques in humans and neural recordings in animals (Shannon 1983; Middlebrooks and Bierer 2002; Snyder et al. 2004; Carlyon et al. 2017). The processing of temporal fine structure (TFS) is also limited. When a single electrode is stimulated at moderate rates, pitch increases with increasing rate but the minimum detectable rate difference is usually substantially larger than for pure tones in NH (Moore and Carlyon 2005). As rate increases further, pitch no longer increases once the rate exceeds an upper limit, which varies between about 200–700 pps depending on the listener and on the electrode stimulated (Townshend et al. 1987; Kong and Carlyon 2009; Carlyon et al. submitted). When bandpass filtered harmonic complexes, designed to minimise place of excitation cues to pitch, are presented to normal hearing listeners the highest upper limit observed is approximately 700 pps (Macherey and Carlyon 2014). This is consistent with the upper limit in the “best” CI listeners being approximately equal to that obtained in NH, but with many CI subjects showing a much lower limit.

A possible neural correlate of the upper limit has been observed in single-neuron recordings in the inferior colliculus (IC) of anaesthetized cats, which phase lock to electrical pulse trains up to a certain rate beyond which they exhibit only an onset response (Snyder et al. 1995; Vollmer et al. 2005; Middlebrooks 2008; Middlebrooks and Snyder 2010; Hancock et al. 2013; Vollmer et al. 2017). Although it is not known whether the limitation arises at or before the IC, there is evidence from humans that the limitations on TFS processing arise centrally to the auditory nerve (AN). We have measured the electrically evoked compound action potential (ECAP) and pulse-rate discrimination in the same subjects, and found good encoding of pulse rate in the ECAPs even at rates where behavioural discrimination was at chance (Carlyon and Deeks 2015).

There is direct evidence that the physiological upper limit of temporal processing is reduced by auditory deprivation. For example, juvenile deafened cats show a higher limit when they have grown up listening through a CI than when they have grown up deaf (Hancock et al. 2013; Vollmer et al. 2017). Indirect evidence from humans, consistent with an effect of auditory deprivation and chronic stimulation on the psychophysical upper limit, comes from the finding in one study that it correlates negatively with the duration of deafness (Cosentino et al. 2016). There is also some evidence, discussed in “Efficacy” under the “Discussion” section, that in both cats and humans, the upper limit following auditory deprivation can be increased by a period of chronic stimulation (Vollmer et al. 2005; Carlyon et al. submitted).

Fine temporal processing in the auditory system relies on the ability of neurons, at and central to the AN, to fire in a sustained and temporally accurate fashion at high stimulus repetition rates (Song et al. 2005). This firing property is dependent on the expression of Kv3 high-voltage-activated potassium channels. Kv3 channels are activated by depolarization of the plasma membrane to potentials above − 20 mV; they open rapidly during the depolarising phase of the action potential in order to initiate repolarisation and prevent significant sodium channel inactivation. As the neuron begins to repolarise, the channels deactivate quickly and thus do not contribute significantly to the after-hyperpolarisation (Rudy et al. 1999; Rudy and McBain 2001). As a consequence, neurons expressing Kv3 channels are able to sustain action potential firing at high frequencies. Kv3.1 and Kv3.3 channel subtypes are expressed in fast spiking neurons throughout the auditory brainstem (Grigg et al. 2000; Li et al. 2001). Loss of Kv3.1 channel expression in the auditory brainstem is associated with ageing (Jung et al. 2005; Zettel et al. 2007) and with auditory deprivation arising from hearing impairment (von Hehn et al. 2004).

AUT00063 is a novel small-molecule drug that selectively enhances Kv3 channel function. In vitro electrophysiology studies with recombinant human Kv3.1 channels expressed in mammalian cells have shown that AUT00063 can increase the amplitude of hKv3.1-mediated potassium currents with a pEC50’s of 5.1 ± 0.17 (Anderson et al. 2018). In addition, two studies showed that AUT00063 reduces the elevation in spontaneous firing rate that results from noise exposure, both in the dorsal cochlear nucleus (“DCN”: Glait et al. 2018) and IC (Anderson et al. 2018). Those studies also showed that AUT00063 can increase neural thresholds (Glait et al. 2018) and reduce driven rates (Anderson et al. 2018) to acoustic stimulation. More importantly for the present study, it has been shown that AUT00063 can improve fine temporal coding in the auditory brainstem in mice. Chambers et al. (2017) exploited their previous finding (Chambers et al. 2016) that Oubain administration, which killed 95 % of AN type 1 neurons, degraded the temporal representation of trains of acoustic chirps in the IC and auditory cortex, and showed that this degradation could be partially reversed by AUT00063, in vivo. Specifically, they demonstrated that AUT00063 increased the precision of phase locking in the IC, particularly at pulse rates faster than about 40 Hz, and improved the accuracy of a classifier that was trained to decode pulse rate from the responses of cells in the IC or auditory cortex. They also showed that, in vitro, AUT00063 reduced the width and increased the precision of action potentials recorded from fusiform neurons of the DCN, which provide a principal input to the IC. Further evidence for the effect of AUT00063 on temporal coding comes from a preliminary report showing that, whereas aged rats exhibit higher gap detection thresholds than younger rats, this elevation can be partially reversed by AUT00063 (Rybalko et al. 2014).

Two previous clinical trials of the effect of AUT00063 on acoustic hearing confirmed the safety of the drug at doses up to 800 mg/day, but revealed no significant effect on either tinnitus or on speech perception in people with hearing loss in older age (Autifony Therapeutics 2014, 2017b). However, acoustic studies of the effects of auditory deprivation, and its possible amelioration by a pharmaceutical agent, are limited by the fact that one can only test patients who have some useable residual hearing. This necessarily excludes those patients who will have experienced most deprivation, namely those who are profoundly deaf. CIs provide an almost unique opportunity to study such patients. Accordingly, Autifony Therapeutics Ltd., who are the inventors of AUT00063, decided to test its effects on hearing among profoundly deaf patients whose hearing was restored by a CI. The initial design of this “QuicK^+^fire” trial tested speech and music perception using stimuli presented via the patients’ clinical processor, and the results of those investigations are described elsewhere (Sanchez et al. 2018). However, as the clinical processors typically remove TFS, and because AUT00063 has been shown to restore the processing of fine temporal information, we decided to evaluate it using direct-stimulation psychophysical experiments that were designed to measure temporal processing by CI users. To do so, we used tests that were well-established in our laboratory and that we had shown to be sufficiently sensitive to modest effects of chronic stimulation and/or stimulus level (Carlyon et al. submitted). The rationale was to maximise the possibility of observing a significant effect by using methods that measure the processing that the drug was designed to improve. If—as turned out to be the case—no significant benefits were found, one could exclude the explanations that either the CI processor removed the appropriate (TFS) information, and/or that the tests were not sufficiently sensitive to reveal a significant effect.

## Methods

### Overview of Protocol and Subject Selection

**Fig. 1:**
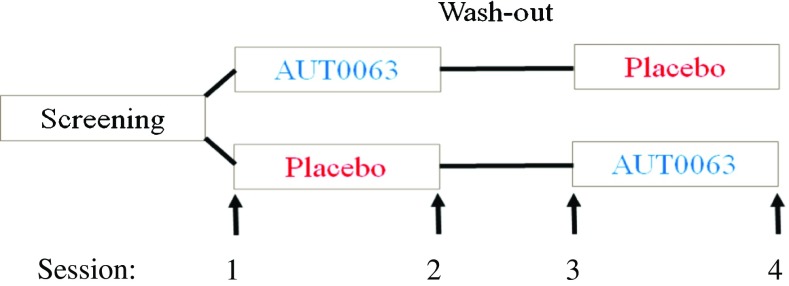
Schematic illustration of the experimental design

A randomised placebo-controlled crossover design was used. Subjects were tested on four occasions, before and after two 28-day periods of taking AUT00063 or a placebo daily, with a 3-week washout period between the second and third testing sessions (Fig. 1). To accommodate the logistics of testing patients from geographically distributed sites, the testing schedule was amended for the tests described here, such that post-dose tests (sessions 2 and 4) could take place at any point between the 20th day after dosing began and the day after the last dose; the actual testing points for the AUT00063 condition are shown for each subject in Table 1. There was no significant difference between AUT00063 and placebo conditions for the average number of days after the initial dose that testing was performed. The AUT00063 dosage consisted of four 200 mg capsules once daily; placebo capsules were visually identical to the drug capsules. Subjects were randomly assigned to two groups, which differed only in the order in which AUT00063 and the placebo were administered.

**Table 1:** Details of the patients who completed the study. “Order” refers to the order of testing, where 1-AUT00063 followed by placebo and 2 = placebo followed by AUT00063. “AUT test day” shows the number days after the first dose on which the tests were performed in the AUT00063 condition. The last two columns show the RMS errors between the broken-stick fit and the data in the upper-limit measures for AUT00063 and placebo, respectively

Subject	Age (years)	Deafness duration (years)	Device	Order	AUT test day	RMSE (AUT)	RMSE (placebo)
S01	76	1.5	Cochlear	1	21	0.43	0.38
S02	58	51.6	Cochlear	1	22	0.31	0.39
S03	78	36.8	Cochlear	1	21	0.31	0.47
S04	73	26.2^a^	Cochlear	1	28	1.10	0.99
S05	69	0.6	Cochlear	1	21	0.76	0.83
S06	63	4.0	Cochlear	2	23	1.27	0.86
S07	63	4.2	Cochlear	2	23	0.66	1.19
S08	79	6.5	Med El	1	23	0.57	0.54
S09	46	30.1	Med El	2	21	0.66	0.84
S10	73	11.2	Med El	1	22	0.22	0.36
S11	68	2.0	Med El	1	22	0.81	0.38
S12	82	1.8	Med El	2	21	0.67	0.73

^a^Duration given for this subject is time since first aided; the time since onset of hearing loss was 43.8 years

Twelve subjects were initially recruited from four clinical sites in the UK, based in London, Cambridge, Manchester, and Birmingham. They received travel expenses and an honorarium for taking part. Inclusion criteria included unilateral implantation with MedEl, Cochlear, or Advanced Bionics devices within the previous 9 to 48 months, BKB audio-only speech scores of between 25 and 95 %, English as first language, and at least 80 % of the electrode array functioning and mapped. A non-exhaustive list of exclusion criteria consisted of subjects with severe tinnitus, those suffering from anxiety or depression, those taking CNS-penetrant medication prohibited by the study protocol, pregnant or nursing women, and people with major diseases that were likely to be jeopardised by entering the study. Further details of the inclusion and exclusion criteria are available at the US public registry and at the European Clinical Trials Register, where the study was registered (Autifony Therapeutics 2017a, c). Three subjects dropped out of the study before completion and were replaced; only the results of the 12 subjects who completed the study are reported here. Because of the blinding it was not possible to ensure that the new subjects had been assigned to the same groups as those they had replaced. Of the final sample, eight were administered AUT00063 followed by placebo, and four with placebo followed by AUT00063. Five were implanted with a MedEl device and seven with a Cochlear device; further information on subjects and device type is given in Table 1.

All methods were described in a testing manual that was prepared by the first author’s laboratory and approved by the sponsor prior to the start of the study. Similarly, all statistical analyses of AUT00063’s safety and efficacy were described in a Statistical Analysis Plan prepared by the Contract Research Organisation, SynteractHCR, according to ICH E9 guidelines and was finalised prior to unblinding of treatment assignments. Additional analyses, for example those concerning test-retest reliability and correlations between the different tests, were performed and specified subsequently by the authors. Following completion of the testing and prior to unblinding of treatment assignments, a blind data review meeting considered any protocol deviations. No substantial deviations, which would have resulted in subjects being excluded from the analysis, were identified.

### Psychophysical Testing

#### Overview

Each testing session lasted approximately 2–3 h and consisted of psychophysical tests performed on an apical electrode of the patient’s device. This was electrode 4 for MedEL users and electrode 20 for Cochlear users. The measures of interest were rate discrimination of pulse trains with rates centred on 120 pps, the upper limit of temporal pitch obtained using a pitch-ranking procedure with pulse rates between 90 and 981 pps, and gap detection thresholds for 1055-pps pulse trains. They are described in detail below.

The same general method was used for all subjects, regardless of the device they were implanted with. In particular, we always used a dB scale to adjust current levels, even though current level is usually specified in linear units for the MedEL device. All stimuli consisted of trains of symmetric cathodic-first biphasic pulses. All pulses had a duration of 43 μs/phase and an inter-phase gap of 0 μs (MedEl) or 8 μs (Cochlear); the difference between these two values is very small relative to the integration time constant of the nerve membrane (e.g. Boulet et al. 2016) and is unlikely to have influenced the results. All stimulation was in monopolar mode; for the Cochlear device both extra-cochlear electrodes were connected in parallel (MP1+2 mode). The same program, written in Matlab, was used to control stimulus presentation and record responses for both devices. The program called low-level routines provided by the respective implant manufacturers as part of the NIC3 (Cochlear) and RIB2 (MedEl) software packages. All stimuli were checked using a test implant and digital storage oscilloscope. In accordance with standard practice in our laboratory, impedances were checked at the start and end of every testing session.

#### Loudness Judgements

It was considered important to equate stimuli in the temporal processing tasks for approximately equal loudness, so as to discourage the use of any potential loudness cues. Each session therefore began with the measurement of most comfortable levels (MCLs) for pulse trains having rates of 90, 162, 399, and 981 pps, in that order. Subjects indicated the loudness of the pulse trains using a chart on which loudness was marked on a scale from 0 (‘off’) to 10 (“too loud”). The experimenter gradually increased the stimulus level until level 7 (“loud but comfortable”) was reached, and then reduced it until the subject indicated a loudness corresponding to point 6 (MCL). After the MCL had been obtained for all rates it was then re-measured for the 90-pps stimulus. The result from this second measurement typically fell within 0.5 dB of that obtained for the first measurement, and was used for the next stage. This consisted of a series of loudness balances, starting with the 90-pps stimulus presented at its MCL, and with the subject adjusting the level of the 162-pps stimulus to be equal in level. This adjusted level was then presented as the fixed stimulus, and the subject adjusted the level of the 90-pps stimulus so as to match its loudness. This was done so as to control for any biases towards under- or over-adjusting the variable stimulus. The difference between the final levels of the two stimuli was averaged for these two stages, and used to determine the final level of the 162-pps stimulus. This procedure was then repeated, matching 399 pps to 162 pps (with the 162 pps pulse train initially fixed at the previously obtained matched level) and then 981 pps to 399 pps. The levels used for all subsequent stimuli in the low-rate discrimination and upper-limit measurements (see below) were obtained by straight-line interpolation of adjacent rates and levels on a log rate vs dB scale.

#### Low-Rate Discrimination

Following loudness estimation we measured rate discrimination thresholds for pairs of pulse trains with rates geometrically centred on 120 pps, using an adaptive procedure (Levitt 1971). In each two-interval forced-choice trial the subject was required to indicate the interval containing the higher pitch, and the response was scored as correct whenever this corresponded to the higher-rate pulse train. Initially subjects performed ten practice trials with pulse rates of 90 and 160 pps. The adaptive procedure started with these pulse rates, and the rate difference was reduced by a factor of 1.25 after every three consecutive correct responses and increased by the same factor after every incorrect response. Stimulus levels were interpolated from the MCL loudness function for each subject. In two cases, both in the first session, the difference between 90 and 160 pps was not discriminable and so caused the lower rate to drop below 90 pps. In those instances the level was set to be the same as that for the 90-pps pulse train. This was done because MCLs vary only slightly with rate decreases below about 100 pps (McKay and McDermott 1998). Correct-answer feedback was provided after every trial. Each change from decreasing to increasing rate difference or vice versa was defined as a turnpoint. The step size was reduced to a factor of 1.1 after the first two turnpoints. The adaptive run ended after eight turnpoints and the threshold, defined as the ratio of the higher and lower pulse rates, was calculated from the geometric mean of the last six turnpoints. The adaptive procedure was then repeated twice. If the standard deviation of the turnpoints for any run exceeded 1.37 an extra run was performed. This criterion was selected as equal to the 95th centile observed in pilot experiments with a different group of subjects. The geometric mean of all three or four rate discrimination ratios (RDRs) was calculated and entered into the analyses.

#### Upper Limit of Temporal Pitch

**Fig. 2:**
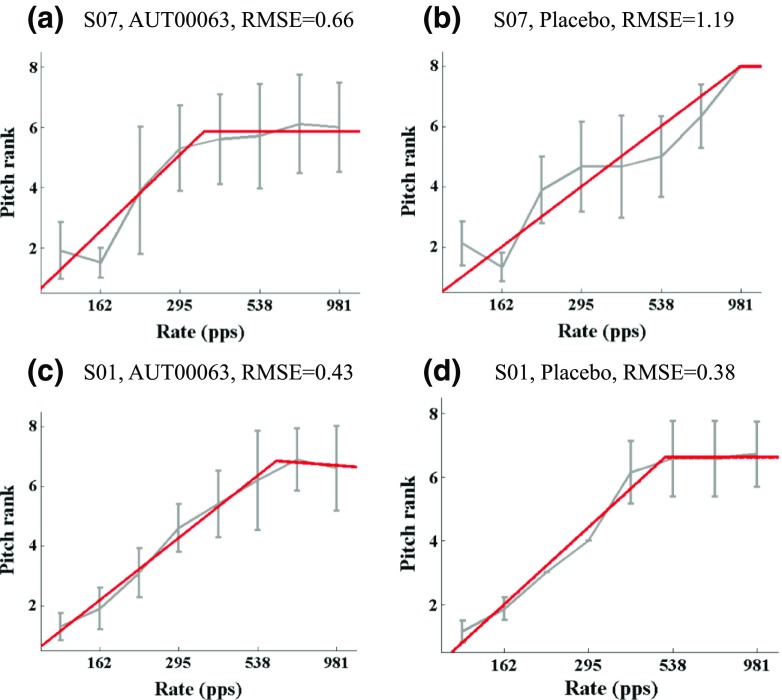
Examples of pitch-ranking functions and the associated broken-stick fits. The subject identifier, condition, and rms error between the broken-stick fit and the pitch ranks are shown at the top of each panel

Following the adaptive procedure listeners pitch-ranked eight pulse rates, equally spaced on a log scale between 120 and 981 pps, using the optimally efficient midpoint comparison procedure (Long et al. 2005). This procedure consists of a series of 2IFC trials without feedback. The procedure was run 10 times, each with the stimuli introduced in a different random order, and the pitch rank for each stimulus was calculated from the mean of these 10 “sub-blocks”. The pitch-rank function was then fit with a “broken stick”, using the Curve Fitting Toolbox from Matlab. The upper limit of pitch was defined as the rate corresponding to the intersection of two straight lines. Examples of four broken-stick fits are shown in Fig. 2. To fit the broken-stick function, the *x*-axis values were first transformed to be between 1 and 8, the number of rates. The constraints were respectively [1, 3] and [− 0.1, 0] for the slopes of the first and second straight lines, [− 10, 1] for the constant term of the first line, and [1, 8] for the *x* value of the intersection between the two lines. Corresponding start values for the fitting procedure were 1, 0, 0 and 4.5. These fitting parameters were selected in advance by inspecting approximately 120 functions obtained in our laboratory from previous experiments, and choosing a set of parameters that yielded upper limits that corresponded well to visual estimates and that were not unduly affected by occasional outliers.

#### Gap Detection

Finally we measured gap detection thresholds (GDTs) for 1033-pps pulse trains, presented at the same level as for the upper-limit measures at a pulse rate of 981 pps. The nominal duration of each pulse train was 400 ms but this was roved by ± 10 % for each stimulus presentation, so as to avoid the use of any duration cues caused by introducing the gap in the signal interval. In each two-interval forced-choice trial listeners discriminated between an uninterrupted pulse train and one with a gap mid-way through. Correct-answer feedback was provided after every trial. At the start of the procedure the gap duration was 40 ms. This was reduced by 40 % after every three consecutive correct answers, and increased by the same percentage after every incorrect answer. The change from decreasing to increasing gap size, or vice versa, defined a turnpoint, and the step size was reduced to 10 % after the first two turnpoints. Each run ended after eight turnpoints, and the geometric mean of the last six was averaged to obtain the GDT for each run. The procedure was then repeated twice. If the standard deviation of the turnpoints for any run exceeded 1.33 ms an extra run was performed. This criterion was selected as equal to the 95th centile observed in pilot experiments with a different group of subjects. The geometric mean of all three or four GDTs was calculated and entered into the analyses.

## Results

### Correlations Between Sessions and Tasks

**Fig. 3:**
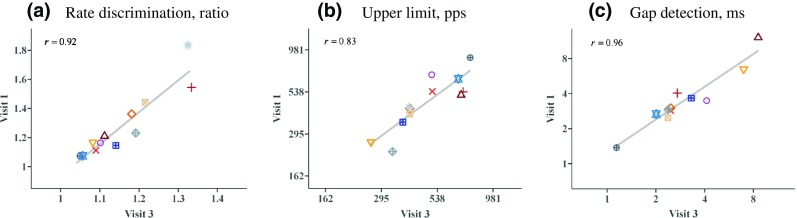
Scatterplots showing performance on visits 1 vs 3 for the **a** low-rate discrimination, **b** pitch ranking, and **c** gap detection tasks

Figure 3 shows the across-subject correlations between the two baseline visits (sessions 1 and 3 in Fig. 1) for the low-rate RDR, upper limit, and GDTs respectively. As with all analyses reported here, the calculations were performed on the (natural) log-transformed scores and plotted using the untransformed scores on a logarithmic scale. Test-retest reliability was high for all three tests, with Pearson correlation co-efficients of 0.94, 0.83, and 0.96 respectively (*t*(10) = 8.71, 4.71, and 10.84, *p* < 0.0001 in all cases). A series of *t* tests revealed no evidence for any change in performance between sessions 1 and 3 for the upper limit (*t*(11) = 0.68, *p* = 0.51). However, thresholds in the other two tests decreased significantly between sessions 1 and 3. The RDR decreased from 1.26 to 1.15 (*t*(11) = 3.38, *p* = 0.006) and the GDT decreased from 3.40 to 2.91 ms (*t*(11) = 3.28, *p* = 0.007). Note that these improvements in the average RDRs and GDTs between sessions 1 and 3 represent learning effects that are not reflected in the test-retest correlations described above. The fact that the RDRs and GDTs improved from sessions 1 to 3 whereas the upper limit did not may be related to the fact that correct answer feedback was provided only for the RDR and GDT tests.

Although each test showed good test-retest reliability with highly significant correlations between sessions 1 and 3, correlations between tests were modest and non-significant. When measures were averaged across sessions 1 and 3, the correlation between the RDR and the upper limit was − 0.20 (*t*(10) = 0.65, *p* = 0.53), that between the GDT and the upper limit was − 0.27 (*t*(10) = 0.89, *p* = 0.40), and that between the GDT and the RDR was − 0.07 (*t*(10) = 0.41, *p* = 0.83).

### Efficacy

For each test, the results from the two post-treatment measures (sessions 2 and 4) were entered into an analysis of variance model with treatment, sequence, subject within sequence, and period as fixed factors (Grizzle 1965). The analysis was performed using the SAS statistical package. In each case the crucial outcomes are the mean and 95 % confidence limits for the effect size, defined as the ratio between the scores in the AUT00063 and placebo conditions. These are shown in bold in Table 2 along with the means for each condition and significance levels for all main effects. Note that good performance corresponds to higher values of the upper limit but to lower values of the RDR and GDT. There was no significant effect of treatment for any measure, and the confidence limits for the treatment effect size all encompass unity.

**Table 2:** Results of the ANOVAs performed to assess the potential effect of AUT00063 on the RDR, upper limit, and GDT. Data in the second and third columns are adjusted for the effects of sequence and period

	Geometric least-squares adjusted mean	95 % confidence (2-sided)	Raw geometric mean	*p* value (2-sided)
RDR				
AUT00063	1.21	1.17–1.26	1.19	
Placebo	1.20	1.17–1.23	1.18	
AUT00063/placebo	1.01	0.96–1.06	1.01	0.63
Period effect				0.47
Sequence (carry over) effect				0.26
Upper limit				
AUT00063	457	349–599	447	
Placebo	491	402–600	480	
AUT00063/placebo	0.93	0.67–1.30	0.93	0.65
Period effect				0.50
Sequence (carry over) effect				0.03
GDT				
AUT00063	2.93	2.56–3.36	2.93	
Placebo	2.96	2.68–3.28	2.93	
AUT00063/Placebo	0.99	0.84–1.17	1.0	0.89
Period effect				0.20
Sequence (carry over) effect				0.98

**Fig. 4:**
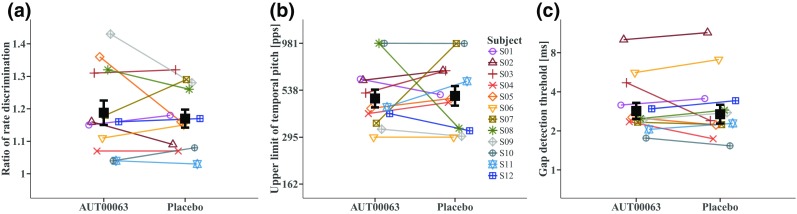
Performance in the AUT00063 and placebo conditions for the **a** low-rate discrimination, **b** pitch ranking, and **c** gap detection tasks. Data for individual subjects are shown by coloured symbols, which are offset horizontally for clarity and joined by coloured lines. The filled black squares and solid black lines show the mean data across subjects, with standard errors indicated by the error bars

**Fig. 5:**
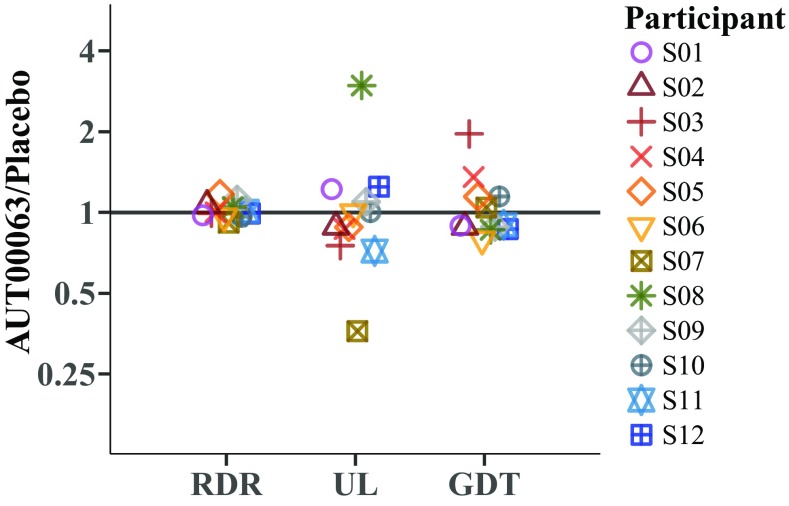
Ratio of scores obtained in the AUT00063 and placebo conditions for low-rate discrimination, pitch ranking, and gap detection. Data for individual subjects are shown by coloured symbols, which are offset horizontally for clarity

The results of the AUT00063 and of placebo conditions are shown for each test in Fig. 4; coloured lines and symbols show data for individual subjects, with mean data indicated by the thick black lines and filled squares. Error bars show standard errors. Figure 5 shows the same data expressed as the ratio of the AUT00063/placebo scores for the rate discrimination, upper limit, and gap detection tests. Both figures illustrate the absence of a significant main effect of treatment. Averaged across subjects, in the placebo conditions the RDR was 1.18, the upper limit was 480 pps, and the GDT was 2.93 ms. The corresponding values after AUT00063 were 1.19, 447 pps, and 2.93 ms. It is also worth noting that, despite the reduction in the RDR and GDT between the two baseline measures (sessions 1 and 3; “Correlations Between Sessions And Tasks” in the “Results” section), there was no significant effect of period in the ANOVAs based on the two post-treatment visits (Table 2).

**Fig. 6:**
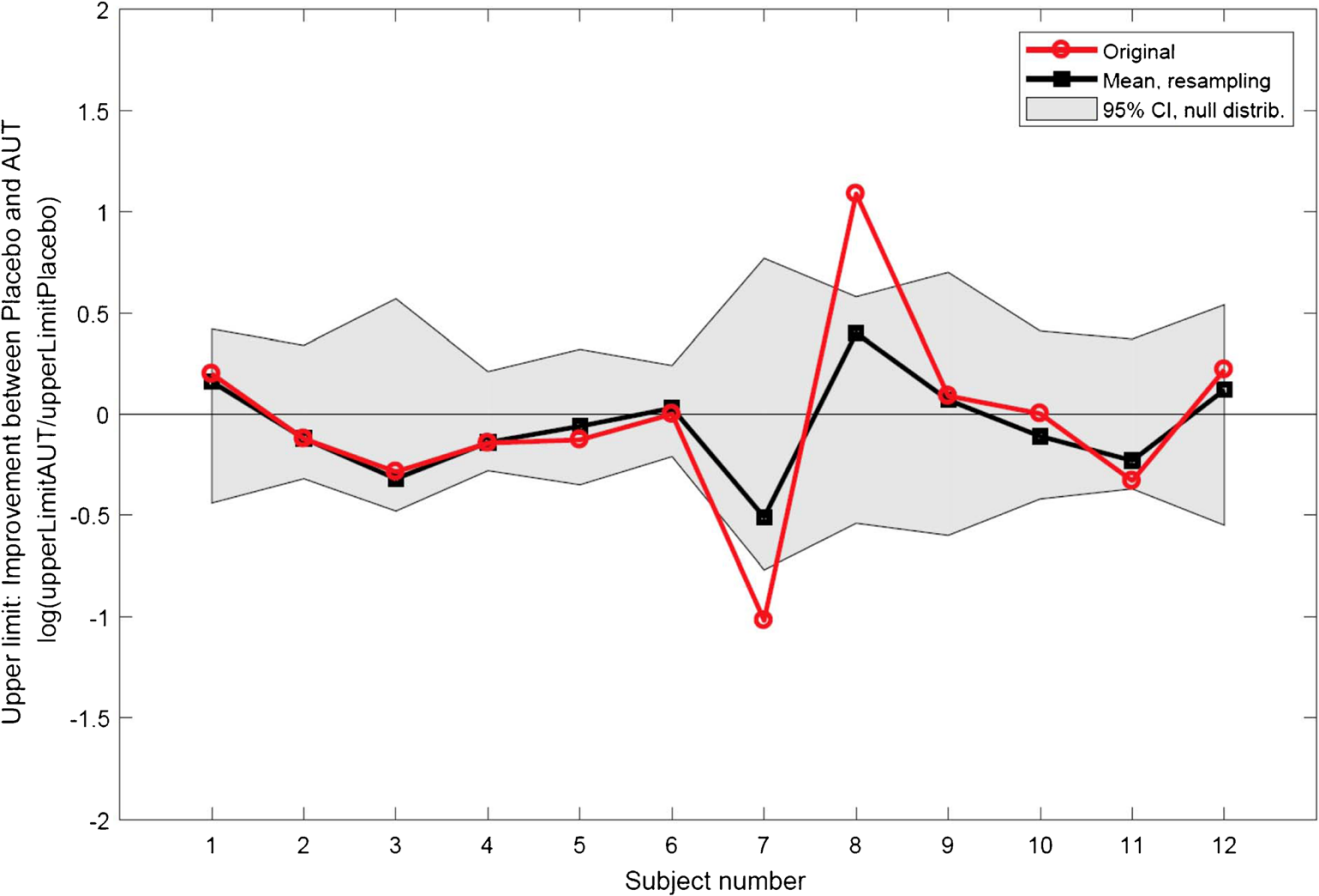
Results of the resampling analysis of the upper-limit data. The ordinate shows the difference between the logarithms of the upper limits in the AUT00063 and placebo conditions (positive values reflect an increase). The solid black line shows the mean of the values obtained for each subject from the broken-stick fits to 200 resamplings of 5 sub-blocks. The shaded area shows the 95 % confidence intervals of the null distribution (see text for details). The solid red line shows the values obtained from our original analysis (Figs. 4 and 5), based on the broken-stick fits to the data from all 10 sub-blocks

It can be seen from Figs. 4 and 5 that two subjects, S07 and S08, showed quite marked and opposite differences between the AUT00063 and placebo tests, and this would have contributed to the relatively large confidence limits for the effect size of the upper limit. Closer examination of the data suggest that the results for subject S07, whose upper limits were 353 pps and 978 pps in AUT00063 and placebo conditions, respectively, were unreliable due to a poor correspondence between the broken-stick function used to estimate the upper limit and the raw data for the placebo condition. That subject’s MPC pitch rank functions are shown with the corresponding fits in Fig. 2a, b for the AUT00063 and placebo conditions respectively. For the AUT00063 condition, which for this subject corresponded to session 4, the fit is reasonably good and the rms error between the function and the raw data was 0.66, which was very close to the average rms error across all subjects and sessions of 0.65. In contrast, the fit for the placebo condition was very poor, and the rms error of 1.19 was the highest for any subject for that session. The rms errors for subject S08 (fits not shown), whose upper limits were 981 and 330 pps in the AUT00063 and placebo conditions, were unexceptional, being 0.54 and 0.57 respectively. In order to investigate whether the lack of a significant treatment effect for the upper-limit measure could be attributed to poor fits, an additional 1-way ANOVA (AUT00063 vs placebo) was performed, in which the cases were weighted by the inverse of the RMS errors. This also showed no significant effect of treatment (*F*(1,20) = 0.019, *p* = 0.89)). Finally, we investigated whether any subject showed a difference between the AUT00063 and placebo conditions at the individual level. To do this, we exploited the fact that, in each of the placebo and AUT00063 sessions, 10 sub-blocks of the MPC were obtained. We first selected, at random, five sub-blocks from each condition and estimated the upper limit from these ten sub-blocks combined. This was then repeated and the difference between the upper limit estimated from the first minus the second sampling was calculated. This whole procedure was then repeated 200 times to obtain the null distribution of differences under the hypothesis of no difference between the sessions; the 95 % confidence limits of this distribution are shown by the shaded area in Fig. 6. We then calculated the observed difference by calculating the between-session difference obtained from five sub-blocks in each condition, again with this procedure repeated 200 times and with the estimated differences averaged. This “true” difference is shown by the solid black line in Fig. 6. It can be seen that the difference for no subject fell outside the 95 % confidence intervals of the null distribution. These differences can be compared to those obtained using our initial analysis based on all ten blocks for each condition (solid red line). It can be seen that, for most subjects, the results of the two analyses are very close. The exceptions are the relatively large differences observed for subjects 6 and 7 in our main analysis (cf. Figs. 4 and 5), which were reduced when estimated using the resampling method.

## Discussion

### Comparisons with Other Studies: Overall Performance and Across-Subject Correlations

The overall size of the RDRs, upper limits, and GDTs reported here are broadly similar to those reported by ourselves and others in studies that did not involve any pharmacological intervention. The mean GDT of 2.9 ms reported here was slightly lower than the average value of 5.1 ms obtained from Advanced Bionics users by Bierer et al. (2015)*,* who used very similar methods to those used here, and falls within the range of GDTs obtained by Garadat and Pfingst (2011) for pulse trains presented at a level corresponding to 90 % of the dynamic range. In a recent study using almost identical methods to those used here, Carlyon et al. (submitted) reported, for nine Cochlear subjects tested 2 months after implant activation, a mean RDR of 1.23 and a mean upper limit of 485 pps. These values are very close to the RDR of 1.18 and upper limit of 480 pps reported here for the placebo condition. The RDRs reported here are also similar to the mean values of 1.22 reported by Cosentino et al. (2016), of 1.16 or 1.28 (depending on electrode) by Stahl & Macherey (2016), and of 1.25 by Bahmer and Baumann (2013).

Some earlier studies have shown smaller RDRs. For example, Moore and Carlyon (2005) analysed the results of 19 listeners from five studies and reported an average RDR of 1.073 at a 100-pps standard rate, although with a large variation across subjects and studies. Goldsworthy and Shannon (2014) showed that extensive training on a rate discrimination task produced significant reductions in RDRs, which, after 6-7 2-hour sessions, were approximately 1.04 at a rate of 110 pps. Learning effects were also observed for the RDRs measured in the present study, as evidenced by the significant difference between sessions 1 and 3 and described in section “Correlations Between Sessions And Tasks” in the “Results” section. The lower RDRs reported in some studies and the presence of learning effects raises the possibility that the larger RDRs reported here and elsewhere do not reveal the “true” limits of temporal sensitivity, and that more central limitations in performance obscured any potential benefit of AUT00063 in the present study. We do not think this is likely because studies that obtained similar RDRs to those observed here have been shown to be sensitive to other manipulations, such as effects of the electrode stimulated (Cosentino et al. 2016; Stahl et al. 2016) and of stimulus level (Carlyon et al. submitted). In addition, we note that the between-session change in RDR observed here was consistent with initial, perhaps procedural, learning during the first session followed by roughly constant performance thereafter: mean RDRs in sessions 1–4 were 1.26, 1.19, 1.15, and 1.16.

As noted in “Correlations Between Sessions And Tasks” in the “Results” section, although performance on each task correlated highly between sessions 1 and 3, between-task correlations were not significant, being − 0.20 between the RDR and upper limit, − 0.27 between GDT and upper limit, and − 0.07 between the RDR and GDT. Those correlations are smaller than reported by Cosentino et al. (2016), which, for the same comparisons, were − 0.47 (t(8) = 1.50, *p* = 0.17), − 0.90 (t(8) = 5.84, *p* < 0.0001), and 0.43 (*t*(8) = 1.35, *p* = 0.21). Cosentino et al. suggested that the significant correlation between the upper limit and GDT might reflect a common neural limitation, associated with sustained and temporally accurate neural responses at high rates, between the two tasks. They argued against a trivial explanation for the correlation, which is that it reflected some non-specific and possibly cognitive between-subject differences, because the correlation between upper limit and GDT was significantly stronger than that between the upper limit and the RDR. The much weaker correlation between upper limit and GDT observed here could be due to the factors limiting performance differing between the two subject groups, or may reflect differences in methods between the two studies. Cosentino et al. (2016) used a different method of estimating the upper limit, and each subject’s scores were obtained from the average of repeated measures on each of four different electrodes. When we combined the *p* values from the two studies using Stouffer’s test, it remained significant at *p* = 0.009.

### Efficacy

The 95 % confidence limits shown in Table 2 impose substantial constraints on the largest possible size of any beneficial effect of AUT00063 for this population and for the dosage size and method used here. The largest improvement due to AUT00063 that fell within the 95 % confidence limits would have been a reduction in the RDR of about 4 %, an increase in the upper limit of about 30 %, and an approximate 16 % reduction in the GDT. The corresponding maximum deleterious effects would have corresponded to 6 %, 33 %, and 17 % respectively.

One possible explanation for the absence of an effect of AUT00063 is that, although our subjects had all undergone extensive periods of auditory deprivation, this was followed by months or years of hearing through their CI, which then restored temporal processing even in the absence of the drug. Vollmer et al. (2005) reported that the upper limit of phase locking in the IC was higher in a group of juvenile deafened cats that had been implanted and stimulated chronically for 21 weeks, compared to an unstimulated group, and was similar to that of a control group that had grown up with normal hearing. However, it appears that in humans, the restorative effects of chronic stimulation are not complete. Carlyon et al. (submitted) measured the psychophysical upper limit and RDR in nine CI users on the day of activation and 2, 6, and 9 months later. The RDR dropped and the upper limit increased significantly between the day of activation (“switch-on”) and 2 months later, but these changes were modest, corresponding to a 36 % increase in the upper limit (95 % confidence interval 20–35 %) and a reduction in the RDR by a factor of 1.06 (95 % confidence interval 1.005–1.210). They noted that these changes may have been partly due to the increases in stimulation level needed at 2 months to maintain the same loudness as at switch-on. Importantly, substantial across-subject differences remained, and the upper limit for most subjects was substantially lower than the 700 pps obtained with NH listeners presented with bandpass filtered pulse trains (Macherey and Carlyon 2014). These across-listener differences have also been shown in one study to correlate with the duration of deafness in a group of subjects who had been using their CIs for months or years (Cosentino et al. 2016).

The demonstrated sensitivity of the RDR and upper limit tests to modest effects of chronic stimulation and level (Carlyon et al. submitted), combined with the good test-retest reliability observed here for all measures, makes it unlikely that the absence of a significant treatment effect can be attributed to low power or to the use of unreliable methods. As noted above, Carlyon et al. (submitted) reported statistically significant changes in the upper limit and RDR from the day of activation to 2 months later, and, regardless of whether those improvements were affected by changes in stimulation level, the study demonstrates the sensitivity of the methods used here. Furthermore, the tests used here were designed to identify improvements in the processing of TFS, via sustained temporally accurate neural firing, which was expected to be the main effect of AUT00063.

Our results contrast with the positive effects observed with acoustic stimulation in animal models and described in the “Introduction”, and particularly with the finding that AUT00063 can improve fine temporal processing in Oubain-treated mice (Chambers et al. 2017). There are several possible reasons for this discrepancy. Considering the dosages used, plasma concentration of AUT00063 was not assessed in the present study; however, the same (800 mg) doses of AUT00063 were administered to subjects with tinnitus in a separate trial (Autifony Therapeutics 2014) and resulted in drug concentrations consistent with those found to be effective in animal models; AUT00063, with mean (median) plasma levels of AUT00063 of 4818.82 (4704.50) ng/ml. However, since this mechanism of drug action has not been explored in humans until now, it is not possible to determine whether humans are more or less sensitive to drugs modulating Kv3 channels in the auditory brain. A notable difference in study design between the present study and the preclinical measures obtained with animal models is the chronic drug administration and testing used here compared to the acute treatment evaluated in successful animal interventions (Rybalko et al. 2014; Chambers et al. 2017; Anderson et al. 2018; Glait et al. 2018). Indeed, there would be considerable value in a future study examining the effects of acute administration of AUT00063 on human hearing. If such an effect were obtained, a useful next step would be to investigate any possible adaptation to the drug. What we can conclude is that any beneficial effect of AUT00063 on temporal processing by human CI listeners, at the dosage used here, is at most small and that we have no evidence that such an effect exists.
